# Optimal threshold of adherence to lipid lowering drugs in predicting acute coronary syndrome, stroke, or mortality: A cohort study

**DOI:** 10.1371/journal.pone.0223062

**Published:** 2019-09-25

**Authors:** Arsène Zongo, Scot Simpson, Jeffrey A. Johnson, Dean T. Eurich

**Affiliations:** 1 School of Public Health, University of Alberta, Edmonton, Alberta, Canada; 2 Faculty of Pharmacy, Université Laval, Quebec City, Quebec, Canada; 3 Population Health and Optimal Health Practices Research Unit, CHU de Québec—Université Laval Research Centre, Quebec City, Quebec, Canada; 4 Faculty of Pharmacy and Pharmaceutical Sciences, University of Alberta, Edmonton, Alberta, Canada; International University of Health and Welfare, School of Medicine, JAPAN

## Abstract

**Objective:**

Thresholds defining medication adherence are rarely evidence-based. A threshold of 0.8 is typically presumed to achieve improved outcomes. We aimed to assess the optimal threshold of adherence to lipid-lowering drugs (LLD) in predicting cardiovascular-related (CV) outcomes in patients with hypertension.

**Design:**

Cohort study of new users of LLDs.

**Setting:**

Comprehensive healthcare administrative databases of the province of Alberta (Canada) from 2008 to 2016.

**Participants:**

Patients with hypertension, who were new users of LLDs. Patients who had the outcomes prior to the initiation of LLD were excluded.

**Main outcomes measures:**

Hospitalization for acute coronary syndrome (ACS)/stroke, CV-related mortality and all-cause mortality.

**Statistical analysis:**

Adherence to LLDs was assessed as the proportion of days covered (PDC) by any LLD, from drug initiation to censoring, outcome, or study end. Three methods were used to assess the threshold: Contal and O'Quigley method, minimum distance method, and Youden's J index. Cox regressions were used to assess the risk associated with each method-specific threshold and Akaike information criteria were used to retain the optimal threshold after adjustment.

**Results:**

52229 patients were included; 4.0% were hospitalized for ACS/stroke, 3.4% died, and 1.3% died from CV-related cause. In predicting ACS/stroke, CV-related and all-cause mortality, the optimal adherence threshold was 0.52 (range: 0.51–0.54), 0.79 (0.45–0.87), and 0.84 (0.79–0.89), respectively. These results were consistent among patients aged ≥ 65 years (n = 19804). However, the results varied among those aged < 65 years, where the incidence rates of outcomes were low.

**Conclusion:**

In new-users of LLDs with hypertension, approximately 50% days covered by LLDs may be enough to prevent long-term occurrence of ACS, or stroke. However, a threshold near 0.80 may be needed to prevent or reduce the risk of all-cause or CV-related mortality.

## Introduction

Hypertension is the biggest single contributor to the global burden of disease and to global mortality, leading to 9.4 million deaths each year [1]. The effect is largely mediated through coronary heart disease and stroke [1]. The proper management of high blood pressure and prevention of coronary disease and stroke are thus a priority.

Dyslipidemia often co-exists in patients with hypertension and is an important risk factor of coronary heart disease and stroke [2, 3], cardiovascular-related death, as well as all-cause mortality [4]. Ongoing management with non-pharmacological (lifestyle modification measures) and pharmacological (lipid-lowering drugs) interventions is required to achieve and maintain target lipid levels [5, 6]. However, poor adherence is common [7], whereby patients rarely use all of their prescribed drugs during the course of the treatment, potentially with serious adverse sequalae.

Although adherence to lipid lowering drugs is essential, it may not be possible for all patients to achieve 100% adherence all the time. This requires using an adherence threshold to distinct patients into good or poor adherers. Although this dichotomization of adherence measure may lead to information loss regarding statistical considerations [8], it is essential in clinical practice for the classification of patients into high or low risk of health outcomes. The level of adherence required to prevent or reduce the risk of cardio-cerebrovascular complications is not well known. A threshold of 0.80 is commonly used to define good and poor adherence, but little is known about the validity (accuracy) of that threshold in preventing adverse health outcomes. In fact, the 0.80 threshold to define good adherence was first set by Sackett and colleagues in 1975 and based on systolic blood pressure response [9]. Since that initial study, the 0.80 threshold has become a kind of standard without much further evidence to support its use. Although few studies have been completed, results suggest that the optimal adherence threshold may vary by nature of disease, treatment, outcomes, and possibly the characteristic of patients [10, 11]. For example, Karve et al. conducted a study aiming to identify the optimal threshold of the proportion of days covered (PDC) by drugs in predicting disease-related hospitalizations in patients with different health conditions. They observed an optimal PDC threshold ranging from 0.58 for congestive heart failure-related hospitalization to 0.85 for diabetes-related hospitalization [10].

Having evidence-based thresholds of medication adherence can contribute to optimal identification of good and poor adherers and properly identify patients in need of intervention. Ultimately, this may result in improved outcomes for patients. Therefore, we undertook the present study to assess the optimal threshold of adherence to lipid-lowering drugs (LLD) in predicting 1) hospitalization for acute coronary syndrome (ACS) or stroke, 2) all-cause mortality and 3) cardiovascular-related mortality in patients with hypertension.

## Methodology

### Study design

We conducted a retrospective cohort study of new users of LLDs using Alberta (Canada) administrative health databases from 2008 to 2016. Patients were followed until outcome, lost to follow-up or study end (maximum of five-year follow-up from the date of LLD initiation).

### Sources of data

We used data from the comprehensive provincial healthcare administrative and vital statistics databases of Alberta Health, Alberta, Canada. These databases maintain current demographic information, and billable services claims including inpatient and outpatient visits and medical procedures, for all patients within Alberta’s universal, publicly funded health care system. Prescription drug dispensations are captured for all Albertans regardless of age, formulary/insurance status and the accuracy and validity are routinely checked through computerized processing. All databases were linked at patient level based on personal health number.

### Study population

The study population comprised patients with hypertension (ICD 9 codes 401.x or ICD 10 codes I11.x) insured by the Alberta Health insurance plan, who were new users of LLD, i.e., had no dispensation of lipid-lowering drugs during an index period of 365 days prior to their first dispensation record of a LLD.

**Fig 1:**
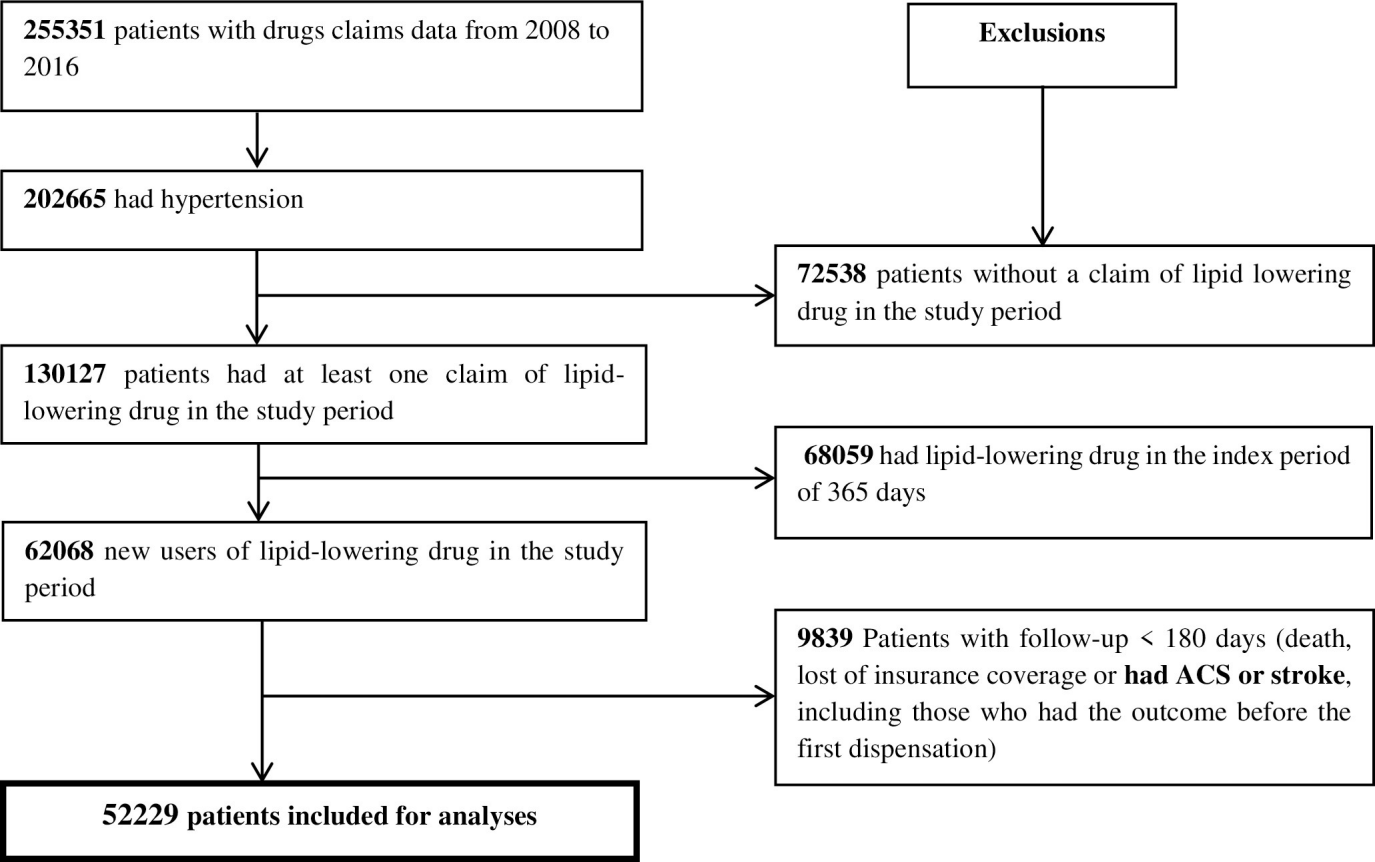
Selection of study population.

We excluded patients with a post-LLD initiation follow-up <180 days (i.e., those who had the outcomes, or out migrated in the 179 days following the first dispensation). This exclusion was necessary to ensure sufficient follow-up time was available to calculate a reasonably accurate estimate of the patients’ adherence. Moreover, clinical trial evidence would further support the notion that LLDs require several months before any beneficial effects are to be realized by patients. We also excluded patients who were hospitalized for ACS or stroke before the initiation of lipid-lowering drugs (Fig 1), to ensure all patients were at the same relative level of risk (i.e., primary prevention patients). Moreover, as ACS and stroke affect future risk of events, exclusion of these patients would further ensure patients were more similar in terms of future risk.

### Adherence to lipid-lowering drugs

We defined adherence based on the proportion of days covered (PDC) by any LLD during the follow-up using drug claims data. Specifically for objective 1 (i.e., outcome of ACS or stroke), we computed the PDC between the first LLD dispensation and the date of ACS or stroke for patients who had the outcome during the follow-up. For patients who were lost to follow-up or having follow-up less than 5 years, we computed the PDC between the first LLD dispensation and the patient specific end date. For the remaining patients who completed follow-up, the PDC was computed in a fixed time window of 1825 days (i.e., a five-year time window). For objectives 2 and 3 (i.e., outcomes of all-cause and cardiovascular-related mortality), we used the same approach as in objective 1 to compute PDC for patients who were lost to follow-up and those having at least five years follow-up. However, for patients who died during the follow-up, we computed the PDC between the first LLD dispensation and the date of death.

The PDCs were adjusted for in-hospital days for patients who were hospitalized during the follow-up (hospital days were subtracted from the denominators) as utilization of LLD during hospitalizations is not captured by the pharmaceutical claims databases and therefore adherence was not calculatable during this period. Switches between different LLD (e.g. simvastatin to lovastatin) were allowed in the PDC calculations.

### Outcomes

Three outcomes were considered to identify the optimal threshold of adherence to LLDs. First, we considered hospitalization for stroke (International Classification of Diseases, 10th Revision [ICD-10] codes: I60, I61, I62, I63, I64) or an acute coronary syndrome (ACS). ACS comprised acute myocardial infarction (ICD-10 codes: I21) angina pectoris (ICD-10 codes: I20) and other ACS (ICD-10 codes: I24). We considered the first event to define the end of the follow-up. These codes have been used in previous studies and are considered to be highly valid in administrative health data [12, 13]. These variables were assessed from the hospitalization registry. Second, we considered all-cause mortality (vital statistics file). The third outcome was cardiovascular-related mortality, defined using ICD-10 codes I00 to I99 for underlying cause of death in the vital statistics file.

We considered these three different outcomes to assess the stability of the optimal threshold as a previous study suggests that the optimal adherence threshold could be outcome-specific [10].

### Other variables

Other variables included age, sex, and comorbidities (defined by ICD-10 codes) at the time of the first dispensation of LLDs. Comorbidities included diabetes, history of ischemic heart disease, heart failure, chronic kidney disease, mental health issue, asthma, and chronic obstructive pulmonary disease (COPD). Finally, we assessed the total number of hospital days during the follow-up.

## Analysis

We used descriptive statistics (proportion, mean or median) to describe the population.

To determine the optimal threshold, two approaches can be considered: breakpoint-based thresholds and classification thresholds [14]. For breakpoint-based thresholds, the relationship between the response (outcome variable) and explanatory variable (i.e., the variable to find the threshold) is not linear, but changes at some point, i.e., the threshold. In contrast, a classification threshold does not necessarily involve a nonlinear relationship between the response and explanatory variable, but instead corresponds to the value of the explanatory variable that optimally classifies the sample into the levels of the response variable [14]. For the present study, three different methods were used to assess classification thresholds, i.e., the threshold of adherence to LLDs that optimally classifies patients into high or low risk profile of ACS or stroke: the Contal and O'Quigley method, the Youden's J index, and the minimum distance method. Contal and O’Quigley's method uses log-rank statistic (Q statistic) to categorize patients into high or low risk groups for the outcome (ACS/stroke or mortality) according to different thresholds of the predictor (i.e., medication adherence). To each threshold a Q statistic is computed and the optimal threshold is selected based on maximizing this Q statistic [15–17].

The minimum distance method and the Youden's J index are derived from receiver operating characteristic (ROC) curve. With the minimum distance method, the optimal threshold value is the cut point associated with the minimum distance from the ROC curve to the upper-left corner of the sensitivity axis (point with coordinates (0, 1)) [18, 19]. If Sn and Sp denote sensitivity and specificity, respectively, the distance between point (0, 1) and any point on the ROC curve is d = √[(1 –Sn)^2^ + (1 –Sp)^2^] [18, 19]. To obtain the optimal threshold, the distances between each of the possible thresholds on the ROC curve and the point (0, 1) were calculated, and the minimum distance was identified [18]. Finally, the Youden's J index is defined as the maximum vertical distance between ROC curve and the diagonal line. It maximizes the difference between True Positive and False Positive. Youden Index Formula J = Sensitivity—(1—Specificity) [18, 19].

After obtaining the thresholds with the three methods, we performed adjusted Cox regressions with PDC dichotomized according to each identified ‘best’ threshold and other closely related thresholds as alternative thresholds. We then used the difference between Akaike Information Criteria (ΔAIC) of models (model with lowest AIC as reference) to retain the optimal threshold. Based on criteria proposed by Burnham and Anderson [20], models with ΔAIC < 4 were retained to define the range of the threshold. Burnham and Anderson proposed that models for which ΔAIC*i* ≤ 2 receive substantial support and should be considered when making inferences, models having 4 ≤ ΔAIC*i ≤* 7 have considerably less support, and models having ΔAIC*i* > 10 receive no support.

In sensitivity analysis, we re-assessed the optimal threshold stratifying the sample according to patients’ age (<65 years and ≥ 65 years) as aging is the largest risk factor for cardiovascular disease [21, 22]. All analyses were carried out using SAS version 9.4 (SAS Institute, Cary, NC, USA).

This research was approved by the Health Ethics Research Board of the University of Alberta (PRO 00060499). All data provided by Alberta Health were fully anonymized. Patients consent was not required to have access to the data.

## Results

In total 52,229 patients were included for analysis (Fig 1). The overall mean age was 61 years (SD 12.5); the majority of patients were men. Mental health issues, diabetes, and ischemic heart disease were the most frequent comorbidities at time of first LLD dispensation (Table 1). During the follow-up (maximum of five years), 2,084/52,229 (4.0%) patients were hospitalized for ACS or stroke, 1,796/52,229 (3.4%) died, and 661/52,229 (1.3%) died from causes related to cardiovascular disease.

**Table 1:** Characteristics of the study population.

Characteristics	Total sample (n = 52229)
Mean age in years (SD)	61.0 (12.5)
Female sex (%)	23990 (45.9%)
Comorbidities at LLD initiation	
Diabetes	22210 (42.5%)
COPD	4871 (9.3%)
Asthma	6687 (12.8%)
Heart failure	3821 (7.3%)
History of ischeamic heart disease	11975 (22.9%)
Mental health issues	26010 (49.8%)
Chronic kidney disease	1879 (3.6%)
Hospitalized during follow-up period (yes vs no)	20127 (38.5%)
Median hospital length of stay during follow-up among hospitalized (n = 20127) (IQR)	8 (3–25)

ACS: acute coronary syndrome; SD: standard deviation; LLD: lipid-lowering drug; IQR: interquartile range

The median PDC was 0.79 (interquartile range: 0.42–0.94) and 0.78 (0.43–0.93), respectively for the ACS/stroke analysis and the mortality analysis.

In predicting ACS or stroke, the Contal and O'Quigley method and the Youden's J index provided the same adherence threshold of 0.52 (Table 2). The minimum distance approach provided a threshold of 0.65. The optimal threshold ranged from 0.51 (ΔAIC = 6.24) to 0.54 (ΔAIC = 3.02) (Table 3) with the threshold of 0.52 providing the lowest AIC. In adjusted Cox proportional hazards models, poor adherence (based on the optimal cut-point of PDC <0.52) was associated with a 69% percent increased risk of an ACS or stroke event (adjusted hazards ratio (aHR) 1.69; 95% confidence interval (CI) 1.54–1.84). Of note, models using the traditional cut point of <0.80 was still associated with increased risk in adjusted Cox models, however, the risk was greatly attenuated (aHR 1.41; 95%CI 1.29–1.54) compared to the optimal cut point of PDC<0.52.

**Table 2:** Optimal thresholds of adherence to lipid-lowering drugs in predicting different outcomes.

Methods to determine the optimal threshold	Outcomes					
	ACS or stroke hospitalization		All-cause mortality		Cardiovascular-related mortality	
	Statistic	PDC threshold	Statistic	PDC threshold	Statistic	PDC threshold
Contal and O’Quigley's method	6.157	0.52	2.943	0.80	1.093	0.47
Youden’s J index method	0.101	0.52	0.060	0.84	0.037	0.79
Minimum distance method	0.650	0.65	0.668	0.79	0.683	0.78

**Table 3:** Comparison of different thresholds of adherence to lipid-lowering drugs (PDC) in predicting 5-year hospitalization for acute coronary syndrome or stroke.

PDC threshold	Adjusted Hazard ratio (95%CI)^¥^	AIC	Difference in AIC (ΔAIC*i*)
PDC < 0.45	1.65 (1.51–1.81)	41831.956	16.854
PDC < 0.50	1.65 (1.51–1.81)	41826.994	11.892
PDC < 0.51	1.67 (1.53–1.82)	41821.344	6.242
PDC < 0.52*	**1.69 (1.54–1.84)**	**41815.102**	**-**
PDC < 0.54	1.67 (1.53–1.83)	41818.118	3.016
PDC < 0.55	1.65 (1.51–1.80)	41824.783	9.681
PDC < 0.65**	**1.61 (1.47–1.75)**	**41831.060**	**15.958**
PDC <0.70	1.52 (1.39–1.66)	41854.867	39.765
PDC <0.80	1.41 (1.29–1.54)	41883.776	68.674
PDC <0.95	1.11 (1.00–1.24)	41939.465	124.363

^¥^Models were adjusted for age, gender, diabetes, COPD, asthma, heart failure, history of ischemic heart disease, mental health issue, chronic kidney disease, and hospitalization.

*Cut-off provided by the Contal and O'Quigley Method (survival analysis) or the Youden index (Area Under ROC curve analysis)

**Minimal distance criterion cut-off

In predicting all-cause mortality, the three methods provided similar thresholds: 0.80 by the Contal and O'Quigley method and the minimum distance approach, and 0.84 by the Youden's J index method (Table 2). The optimal threshold ranged from 0.79 to 0.89 (Table 4) with the threshold of 0.84 providing the lowest AIC (Table 4). In adjusted Cox proportional hazards models, poor adherence (based on the optimal cut-point of PDC <0.84) was associated with a 39% percent increased risk of mortality (aHR 1.39; 95%CI 1.26–1.52). Models using a more traditional cut point of <0.80 provided similar results (aHR 1.37; 95%CI 1.24–1.50).

**Table 4:** Comparison of different thresholds of adherence to lipid-lowering drugs (PDC) in predicting 5-year all-cause mortality.

PDC threshold	Adjusted Hazard ratio (95%CI)^¥^	AIC	Difference in AIC (ΔAICi)
PDC < 0.75	1.31 (1.19 1.43)	33897.279	13.703
**PDC < 0.79**^**£**^	**1.35 (1.23–1.49)**	**33888.656**	**5.080**
PDC < 0.80*	**1.37 (1.24–1.50)**	**33886.078**	**2.502**
PDC < 0.84**	**1.39 (1.26–1.52)**	**33883.576**	**-**
PDC < 0.89	1.39 (1.26–1.54)	33886.895	3.319
PDC < 0.90	1.36 (1.23–1.51)	33893.001	9.425
PDC < 0.95	1.24 (1.10–1.40)	33915.741	32.165

^¥^Models were adjusted for age, gender, diabetes, COPD, asthma, heart failure, history of ischemic heart disease, mental health issue, and chronic kidney disease.

^£^ Cut-off provided by the Minimal distance method

* Contal and O'Quigley Method cut-off (survival analysis)

** Youden index method cut-off

In predicting cardiovascular-related mortality, the thresholds provided by the three methods were more variable with estimates of 0.47, 0.78, and 0.79, respectively by the Contal and O'Quigley method, the minimum distance approach, and the Youden's J index (Table 2). The optimal threshold ranged from 0.45 to 0.87 (Table 5) with the threshold of 0.79 providing the lowest AIC (Table 5). In adjusted Cox proportional hazards models, poor adherence (based on the optimal cut-point of PDC <0.79) was associated with a 25% percent increased risk of mortality (aHR 1.25; 95%CI 1.07–1.45), essentially identical to a more traditional cut point of <0.80 (aHR 1.24; 95%CI 1.06–1.45).

**Table 5:** Comparison of different thresholds of adherence to lipid-lowering drugs (PDC) in predicting 5-year cardiovascular-related mortality.

PDC Cut-off	Adjusted Hazard ratio (95%CI)^¥^	AIC	Difference in AIC (ΔAICi)
PDC < 0.40	1.15 (0.96–1.37)	12623.098	5.471
PDC < 0.45	1.20 (1.01–1.42)	12621.167	3.540
**PDC < 0.47**^**£**^	**1.23 (1.04–1.45)**	**12619.995**	**2.368**
PDC < 0.70	1.15 (0.99–1.35)	12622.171	4.544
PDC < 0.75	1.19 (1.02–1.38)	12620.743	3.116
PDC < 0.78*	**1.24 (1.06–1.44)**	**12618.126**	**0.499**
PDC < 0.79**	**1.25 (1.07–1.45)**	**12617.627**	-
PDC < 0.80	1.24 (1.06–1.45)	12618.005	0.378
PDC < 0.85	1.21 (1.03–1.41)	12619.881	2.254
PDC < 0.87	1.15 (0.98–1.35)	12622.321	4.694
PDC < 0.90	1.12 (0.95–1.32)	12623.467	5.840
PDC < 0.95	1.04 (0.86–1.26)	12625.273	7.646

^¥^Models were adjusted for age, gender, diabetes, COPD, asthma, heart failure, history of ischemic heart disease, mental health issue, and chronic kidney disease.

^**£**^Cut-off provided by the Contal and O'Quigley Method (survival analysis)

* Cut-off provided by the Minimal distance method

** Cut-off provided by the Youden index method

In sensitivity analysis among the 65 years or over, the Contal and O’Quigleys’s method and the Youden’s J index method provided the same threshold of 0.65 for ACS/stroke, while the Minimum distance method provided 0.75. In predicting all-cause mortality, the thresholds were 0.81, 0.84, and 0.88, respectively with the Minimum distance method, the Contal and O’Quigleys’s method and the Youden’s J index which was similar to the main analyses. For cardiovascular-related mortality, the three methods yielded the same threshold of 0.79 (see S1 Table) identical to that observed in the main analysis.

Among the patients < 65 years, the Contal and O’Quigleys’s method and the Youden index also provided a same threshold of 0.53 for ACS/stroke, while the Minimum distance method provided 0.63. The same threshold of 0.80 was observed for all-cause mortality as the main analysis. For cardiovascular-related mortality, the thresholds were 0.66, for the Youden’s J index method and the Minimum distance method, and 0.96 for the Contal and O’Quigley's method (see S2 Table).

## Discussion

Our study demonstrated that optimal threshold of adherence to LLD in predicting hospitalization for ACS/stroke, all-cause mortality or cardiovascular-related mortality may be different among patients with hypertension. Indeed, we observed an optimal threshold of 0.52 in predicting hospitalization for ACS/stroke and a threshold around 0.80 for all-cause and cardiovascular-related mortality. Our finding related to all-cause mortality were robust among patients below or above 65 years. However, in predicting ACS/stroke in patients aged 65 years or more, a slightly higher threshold of 0.65 was observed compared to 0.52 for the overall sample.

In predicting ACS/stroke, we observed that models with thresholds higher than 0.52 were associated with a relatively poor model fit. This suggests that using a threshold of 0.80, as is commonly used, could lead to a less efficient assessment and/or misclassification bias of the relation between adherence to lipid-lowering drugs and hospitalization for ACS/stroke.However, our results suggest that the threshold to predict ACS/stroke could be higher than 0.52 in patients who are at higher risk of cardiovascular disease. For example, in the sample of patients aged 65 years or older, we observed a threshold of 0.65 with both the Contal and O’Qickley method, and the Youden Index, and 0.75 with the minimum distance approach. We acknowledge that additional stratification with other risk factors would have been useful to further assess the stability of the adherence threshold in predicting these outcomes. However, due to the relatively low event rate, this stratification would have been at the expense of the accuracy and precision of the identified thresholds as we observed in the strata of patients aged under 65 years (threshold to predict cardiovascular-related mortality varied from 0.66 to 0.96). On the other hand, our results on the prediction of mortality (all cause or cardiovascular-related) are consistent with the use of a threshold of 0.80 which has historically been used in adherence research. Indeed, in both the total sample and the subgroup patients at higher cardiovascular risk (i.e., 65 years or older), we observed similar thresholds in predicting all-cause mortality (0.84) and cardiovascular -related mortality (0.79). This suggests a stability of the optimal threshold, i.e., the threshold is less sensitive to patients’ characteristics when predicting these two broader outcomes. Further studies would be necessary to confirm this finding.

Overall, our results suggest that for more specific outcomes like hospitalization for ACS/stroke, a lower threshold of adherence to lipid-lowering drugs below 0.80 could be considered and for broader outcomes, such as all-cause mortality, a higher threshold is needed to identify good and poor adherers. Indeed, when evaluating more broader outcomes like all-cause mortality, it is likely that adherence is a marker of overall healthy behaviours. Our results also emphasized the necessity of conducting sensitivity analysis by varying the threshold of medication adherence as the optimal threshold may not be a single point for all patients and for all outcomes. Indeed, our sensitivity analysis according to patients’ age provided different thresholds to some extent.

The varying nature of the optimal adherence threshold we observed was consistent with results of previous studies. Indeed, Karve et al. in their study of patients treated with monotherapy for schizophrenia, hypertension, hyperlipidemia, diabetes, or congestive heart failure, reported optimal thresholds of PDC varying from 0.58 in predicting congestive heart failure-related hospitalization to 0.85 in predicting diabetes-related hospitalization. The authors also observed that the optimal threshold to predict all-cause hospitalization in each group of patients was different from those of the disease-related hospitalization [10]. Lo-Ciganic et al. also observed optimal adherence thresholds varying from 0.46 to 0.94 according to patient health and medication complexity in predicting all-cause hospitalizations among patients with diabetes [11]. Therefore, the use of a single standard threshold to identify good or poor adherers should be reconsidered.

Strengths of our study include the large sample size and long-term follow-up. We also considered three different outcomes to assess the possible variation or stability of the optimal adherence threshold. Finally, we used three different approaches to identify the thresholds and survival regression analyses to compare the identified thresholds to other thresholds and to retain the range of each optimal threshold. Our study had some limitations, however. First, we computed adherence using drug claims data that assumes that drugs refilled by patients are properly used. This assumption may overestimate adherence to their treatment for some patients. However, the effect of this possible bias on optimal adherence threshold is difficult to predict. Second, the small number of cardiovascular-related deaths in the total sample (1.30%) may have impacted the accuracy of the threshold in predicting this outcome. The broad range of the optimal threshold for this outcome in the total sample (0.45 to 0.87) and the large difference between the threshold obtained with the Contal and O’Quigleys’s method (0.47) and the two other methods (0.78 and 0.79) tend to support this possibility. However, among patients aged 65 years or above, the number of cardiovascular-related deaths was higher (2.56%) and allowed for improved accuracy of the threshold in predicting this outcome (all the three methods provided the same threshold of 0.79) suggesting that regardless of age, 0.79 is a suitable cut point. In general, the relative small number of events did not allow further stratification of the analyses by combining patients’ characteristics (for example, strata of male patients aged 65 years or more, with diabetes or other strata would be interesting to assess). Future studies that include more individuals and longer follow-up would be necessary to assess the stability of the thresholds in different CV risk groups. Finally, our regression analysis did not account for all potential confounders. However, this issue is less likely to have a significant effect on the comparison of the thresholds as it is likely to affect each model performance equally.

In conclusion, our results suggest that the threshold to identify poor and good adherers to lipid-lowering drugs should be outcome-specific. In assessing the relationship between adherence to lipid-lowering drugs and ACS/stroke, a threshold lower than the frequently used 0.80 threshold may be worthy of consideration. However, the 0.80 threshold may be most suitable for predicting mortality. Finally, varying the threshold in sensitivity analysis should be required in studies of adherence to capture the overall relationships between medication adherence and these outcomes.

## Disclaimer

This study is based in part on data provided by Alberta Health. The interpretation and conclusions contained herein are those of the researchers and do not necessarily represent the views of the Government of Alberta nor the funder (Institute of Health Economics). Neither the Government nor Alberta Health nor the Institute of Health Economics express any opinion in relation to this study.

## Supporting information

S1 Table Optimal thresholds of adherence to lipid-lowering drugs in predicting different outcomes among hypertensive patients aged ≥ 65 years. (DOCX)

S2 Table Optimal thresholds of adherence to lipid-lowering drugs in predicting different outcomes among hypertensive patients aged < 65 years. (DOCX)
