# Supplementary material for: Optimal threshold of adherence to lipid lowering drugs in predicting acute coronary syndrome, stroke, or mortality: A cohort study
Source: PLoS One. 2019 Sep 25;14(9):e0223062. doi: 10.1371/journal.pone.0223062 (PMC6760888; doi:10.1371/journal.pone.0223062)
Supplement: S1 Table — (DOCX) [file pone.0223062.s001.docx]

**S1 Table. Optimal thresholds of adherence to lipid-lowering drugs in predicting different outcomes among hypertensive patients aged ≥ 65 years**

| Methods to determine the threshold | Outcome | | | | | |
| --- | --- | --- | --- | --- | --- | --- |
|  | ACS or stroke  [1081/19804 (5.46%)] | | All-cause mortality  [1339/19804 (6.76%)] | | Cardiovascular-related mortality  [506/19804 (2.56%)] | |
|  | Statistic | PDC threshold | Statistic | PDC threshold | Statistic | PDC threshold |
| Contal and O’Quigley's method | 2.963 | 0.65 | 4.23 | 0.84 | 2.135 | 0.79 |
| Youden’s J index method | 0.077 | 0.65 | 0.102 | 0.88 | 0.087 | 0.79 |
| Minimum distance method | 0.665 | 0.75 | 0.639 | 0.81 | 0.645 | 0.79 |
