# Supplementary material for: Optimal threshold of adherence to lipid lowering drugs in predicting acute coronary syndrome, stroke, or mortality: A cohort study
Source: PLoS One. 2019 Sep 25;14(9):e0223062. doi: 10.1371/journal.pone.0223062 (PMC6760888; doi:10.1371/journal.pone.0223062)
Supplement: S2 Table — (DOCX) [file pone.0223062.s002.docx]

**S2 Table. Optimal thresholds of adherence to lipid-lowering drugs in predicting different outcomes among hypertensive patients aged < 65 years**

| Methods to determine the threshold | Outcome | | | | | |
| --- | --- | --- | --- | --- | --- | --- |
|  | ACS or stroke  [1003/32425 (3.09%)] | | All-cause mortality  [457/ 32425 (1.41%)] | | Cardiovascular-related mortality  [155/32425 (0.48%)] | |
|  | Statistic | PDC threshold | Statistic | PDC threshold | Statistic | PDC threshold |
| Contal and O’Quigley's method | 5.198 | 0.53 | 1.360 | 0.80 | 1.030 | 0.96 |
| Youden’s J index method | 0.145 | 0.53 | 0.070 | 0.80 | 0.070 | 0.66 |
| Minimum distance method | 0.610 | 0.63 | 0.669 | 0.80 | 0.680 | 0.66 |
